# Kuntai Capsule Inhibited Endometriosis via Inducing Apoptosis in a Rat Model

**DOI:** 10.1155/2016/5649169

**Published:** 2016-08-11

**Authors:** Ruihua Zhong, Aying Ma, Jianping Zhu, Guoting Li, Shuwu Xie, Zhao Li, Youlun Gui, Yan Zhu

**Affiliations:** ^1^Department of Reproductive Pharmacology, Key Laboratory of Reproduction Regulation of NPFPC, SIPPR, IRD, Fudan University, Shanghai 200032, China; ^2^School of Pharmacology, Guangdong Medical College, Dongguan 523808, China

## Abstract

We evaluated the effectiveness of Kuntai Capsule (KTC) for treating endometriosis using rat model and investigated its preliminary mechanism of action involved. SD rats were implanted with endometrial tissues and treated with KTC for three weeks. Then, laparotomy was performed to examine volume changes of the autografts. The serum levels of TNF-*α*, IL-6, COX-2, E_2_, and P4 were measured through ELISA. TUNEL was performed to analyze the apoptosis on ectopic endometrium. Protein levels of caspases 8, 9, and 3 and cytochrome c in the ectopic and eutopic endometrium were measured by western blotting. Results showed that KTC significantly decreased the volumes of ectopic endometrium. The level of TNF-*α* increased and E_2_ decreased in the KTC treatment groups. TUNEL and western blot assay showed that KTC could induce apoptosis of endometriotic tissues, accompanied with the increased protein expression of caspases 8 and 9, activated caspase-3, and cytochrome c in a dose-dependent manner. However, these protein expression profiles were not affected in eutopic endometrium. Our findings suggest that KTC could inhibit the growth of ectopic endometrial tissue through upregulating the level of TNF-*α* and its downstream signaling, including caspases and cytochrome c.

## 1. Background

Endometriosis (EM) is a very common debilitating disease affecting 6–10% of women worldwide [1]. Approximately 35–50% of women with endometriosis experience abdominal pain, infertility, or both [1]. However, the etiology of endometriosis remains unclear [2, 3]. Although laparoscopic surgery is now regarded as the best way to remove the endometriotic lesions [4, 5], there are still a great deal of debates on the indication and the outcome of the surgical treatment, such as recurrence, contraindications, postoperative complications, and possible adverse effects on fertility [6–9]. It has been shown that hormonal medication, including progestin, oral contraceptives, and gonadotropin-releasing hormone analogs (GnRHa) are effective in reducing endometriosis-induced pain and decreasing disease recurrence [10, 11]. They are considered as the first-line drugs to treat endometriosis [12]. Besides these chemical compounds, various low-toxic herbs have also been demonstrated to attenuate the gynecological diseases in China [13, 14]. It is worth investigating the efficacy and mechanism action of Chinese traditional herb medicine in relieving the symptom of endometriosis.

Kuntai Capsule (KTC), a type of herb formulas, was first described in the book of* Shang Han Za Bing Lun* in the 3rd century. The formula of KTC consists of six traditional Chinese herbs, including radix* rehmanniae preparata, Scutellaria baicalensis, Coptis chinensis *Franch.,* Poria cocos *(Schw.) Wolf,* white peony root*, and* gelatin.* The formula has been used to improve women's menopause-related symptoms in China [15]. Previous study has shown that KTC could apparently reduce the incidences of vaginal bleeding and breast distending pain compared to Tibolone when combined with GnRH analogs in EMs patients [16]. Tibolone, as a selective tissue estrogen activity regulator, has weak estrogenic activity after metabolizing 3*α*-OH and 3*β*-OH derivatives. Zhang and Li [17, 18] also reported that KTC could improve patients' life quality during the early stage of menopause and alleviate breast pain and vaginal bleeding compared with that in estrogen treatment group.

Although the clinical efficacy of KTC has been widely acceptable in China, its effect on endometrial thickness has not yet been clarified and there is no report on the treatment of endometriosis. We applied the McPhail model which is a traditional classical method to assessment of the progesterone receptor agonist and antagonist activity of a compound. This model can help us to evaluate whether KTC has progesterone activity or not. From our pilot study, KTC could demonstrate a weak progesterone-stimulating effect on immature rabbits (data not shown). Considering the therapeutic benefit of progestin on ectopic endometrium [19], we assumed that KTC could be employed to ameliorate the growth of endometriosis. Therefore, in the present study we established a rat model of endometriosis to evaluate the effect of KTC on the growth of ectopic and eutopic endometrium and preliminarily investigate the underlying mechanism.

## 2. Materials and Methods

### 2.1. Reagents

KTC (lot number 130705) is a product of Guiyang Xintian Pharmaceutical Limited Company (Guiyang, Guizhou province, China). The estradiol (E_2_), progesterone (P), interleukin-6 (IL-6), cancer antigen (CA125), and cyclooxygenase-2 (COX-2) enzyme-linked immunosorbent assay (ELISA) kits were purchased from Nanjing Jiancheng Bioengineering Institute (Nanjing, Jiangsu province, China). The tumor necrosis factor-alpha (TNF-*α*) ELISA kit was purchased from R&D Systems, Inc. (Minneapolis, USA). BCA Protein Assay Kit (P0012S) was bought from Beyotime Biotechnology (Shanghai, China). Rabbit polyclonal caspase-8 (ab25901), caspase-9 (ab2324), cytochrome c (ab90529), and active and procaspase-3 (ab13847) were bought from Abcam Biotechnology (Cambridge, USA). The terminal deoxynucleotidyl transferase dUTP nick-end labeling (TUNEL) kit (11684795910) was purchased from Roche (Basel, Switzerland). An enhanced chemiluminescence (ECL) detection kit was purchased from Boster (Wuhan, Hubei province, China).

### 2.2. Animals

Seventy-four female Sprague-Dawley rats (body weight 220–240 g) were purchased from Sino-British Experiment Animal (Shanghai, China). The experiment was approved by the Laboratory Animal Ethics Committee at the Shanghai Institute of Planned Parenthood Research (Approval number 2015-05). Animals were housed under a 12-h/12-h light/dark cycle and fed with standard chow and water* ad libitum*. Each animal was weighed weekly.

### 2.3. Establishment of Endometriosis in a Rat Model

Sixty rats were used to establish the rat model of endometriosis which was described in previous studies [20, 21] with modification. Briefly, before the first laparotomy operation to confirm that all rats were in estrus, rats were subcutaneously injected with 10 *μ*g/kg of estradiol benzoate per day for two consecutive days before the first laparotomy operation to confirm that all rats were in estrus. The rats were anesthetized with 3% pentobarbital sodium and a small incision was made in the middle of the abdomen to perform the first laparotomy. A segment of the left uterine tissue approximating one centimeter was cut out with surgical scissors. The endometrium was carefully separated from the underlying muscle. Then a portion of the endometrium about 5 × 5 mm was stitched to the right parietal abdominal wall of the rat. The incision was sutured with 4-0 medical suture line. Due to endometriotic lesion growth depending on estrogen [22], estradiol benzoate (30 *μ*g/kg) was then subcutaneously injected on the 1st day and 10th day later to promote the growth of the allograft [21]. After 21 days of recovery the second laparotomy was performed. Three dimensions of the endometrial allograft were measured by vernier calipers. The endometrial volume was calculated applying the prolate ellipsoid formula: *V* (mm^3^) = 0.52 × *A* × *B* × *C*, where *V*, *A*, *B*, and *C* denote volume, width, length, and height in millimeters, respectively [23]. Rats with an endometrial volume exceeding 25 mm^3^ were regarded as successful models of endometriosis. A total of 56 rats were qualified to be used in the following experiments.

The other 14 rats were served as sham-operated group. A segment of fat from the left uterine tissue was stuck to the right parietal abdominal wall of rat. The sham-operated rats were performed laparotomy alone without being measured at second laparotomy.

### 2.4. Treatment with KTC 

The 56 rats were randomly divided into control, leuprorelin acetate, KTC low dose [KTC (L)], and KTC high dose [KTC (H)] groups based on the volume of the autografts. Each group consisted of 14 animals and there was no significant difference on the volume of autografts before solvent and medication treatment. The other fourteen rats with sham-operated group were regarded as negative group. As is shown in Table 1, rats in the sham-operated and control group were given an equal volume of solvent (0.5% carboxymethyl cellulose sodium, CMC-Na). Rats in the leuprorelin acetate group were considered as positive control and treated with 0.1 mg/kg leuprorelin acetate only once by subcutaneous injection according to previous study [24]. Rats in the KTC (L) and KTC (H) group were given 0.24 and 0.6 g/kg/day of KTC by oral gavage, respectively. KTC was suspended in solvent. Animals were given KTC by gavage orally once daily for three consecutive weeks accompanied with vaginal smear. A drop of vaginal washings was placed on a glass slide and examined by light microscopy to observe estrous cycle.

The third laparotomy was performed after solvent and medication treatment according to our previous report [20]. Briefly, the rats were euthanized at the same phase of the estrous cycle. Then the volumes of ectopic endometrium were measured by vernier calipers again. Three ectopic endometrial tissues of each group collected were immediately put into 4% paraformaldehyde solution and the remaining eleven ectopic endometrial tissues were stored at −80°C. The serum samples from the abdominal aortic blood (about 5 mL) were stored at −20°C. The growth inhibitory rate of the ectopic autographs was calculated using the prolate ellipsoid formula: % = (1 − *V*_post_/*V*_pre_) × 100%. *V*_pre_ and *V*_post_ meant the volume of ectopic endometrium before and after treatment, respectively.

### 2.5. Hematoxylin-Eosin Staining

The endometrial tissues were embedded in paraffin and sliced into 4 *μ*m thick sections and stained with hematoxylin-eosin (H&E) for histological examination under a light microscope. Ectopic endometrial thickness was measured by the Motic analysis software.

### 2.6. Measurement of E_2_, Progesterone, IL-6, COX-2, and TNF-*α*

Serum levels of E_2_, progesterone, IL-6, COX-2, and TNF-*α* were assayed by ELISA following the manufacturer's instructions and using a BioTek Synergy 2 Multi-Mode Microplate Reader (BioTek Instruments, Winooski, VT) at wavelength of 450 nm.

### 2.7. TUNEL Assay

A standard protocol for TUNEL was provided by the manufacturer. Paraffin-embedded tissue sections were washed in xylene twice for 5 min each, followed by hydration with a series of 100%, 95%, 85%, and 75% ethanol solutions and ddH_2_O twice for 3 min each. Then the tissue slides were incubated with proteinase K for 30 min at 37°C. After being washed twice with phosphate-buffered saline (PBS), the tissue slides were incubated with 50 *μ*L TUNEL reaction mixture for 1 hour at 37°C in a humidified dark slide box. Then the slides were washed with PBS three times for 5 min each time. The ratios of apoptosis were examined under a fluorescence microscope by counting positively stained cells in four different visual fields selected randomly.

### 2.8. Western Blot Analysis

Proteins were extracted from endometriotic lesions and eutopic endometrium, respectively. Western blot analysis was performed as previously described [23]. The protein concentration was determined using bicinchoninic acid (BCA) method according to the manufacturer's instructions. 80 *μ*g proteins from each sample were loaded onto gels acrylamide: bisacrylamide (29 : 1) for 12% sodium dodecyl sulfate-polyacrylamide gel electrophoresis (SDS-PAGE). The primary antibody dilutions were 1 : 1500 for antibodies of caspase-9, caspase-3, and *β*-actin, 1 : 2000 for caspase-8, and 1 : 500 for cytochrome c. The relative protein levels were semiquantitatively determined by the ratio with *β*-actin using Image Lab software (version 4.0, Bio-Rad Laboratories, Inc., America). Protein levels of five animals per group were measured, respectively.

### 2.9. Statistical Analysis

All data were analyzed with SPSS 17.0 statistics software (version 17.0.0.0, Chicago, IL, USA). Comparisons of variables of interest among groups were performed by one-way analysis of variance (ANOVA) and followed by least squares difference (LSD) tests or Dunnett *t*3-tests. Data are presented as mean ± standard deviation or error. A level of *p* < 0.05 was considered statistically significant.

## 3. Results

### 3.1. The Influence of KTC Treatment on Rat Body Weight and Estrous Cycle

**Figure 1:**
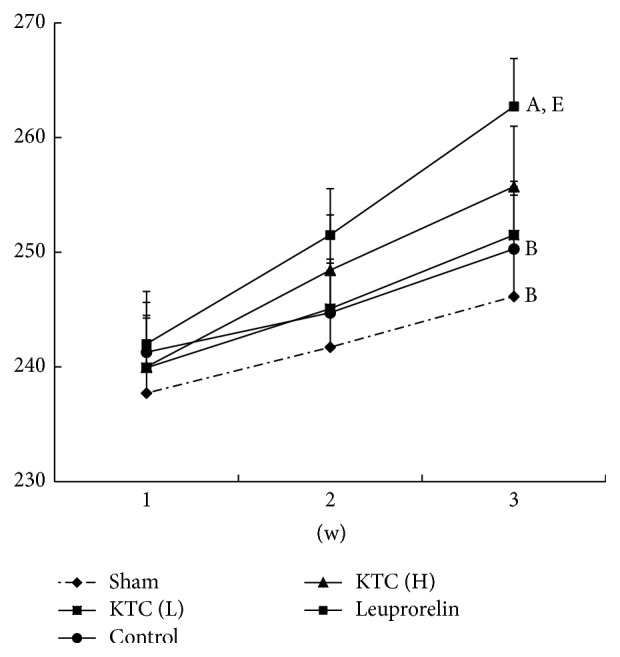
Effect of KTC on the changes of rat body weights (*n* = 14, mean + standard error). ^A^ *p* < 0.05, compared with the control group. ^B^ *p* < 0.05, compared with the leuprorelin group. ^E^ *p* < 0.05, compared with sham-operated group.

The changes of rat body weights were analyzed each week during KTC treatment. As shown in Figure 1, rats treated with either dose of KTC (0.24 or 0.60 g/kg) showed no significant difference compared with that of control group during three weeks (*p* > 0.05). However, the body weight of rats treated with leuprorelin acetate significantly increased compared to those in the control group on the third week (*p* < 0.05).

The vaginal smears of rat were taken to assess the estrous cycle. The results indicated that estrous cycles of rat were routine in sham, control, and KTC groups. However, leuprorelin acetate affected estrous cycle and the rat was at diestrus after treatment for two weeks.

### 3.2. KTC Reduces the Volume of the Ectopic Endometrium

**Figure 2:**
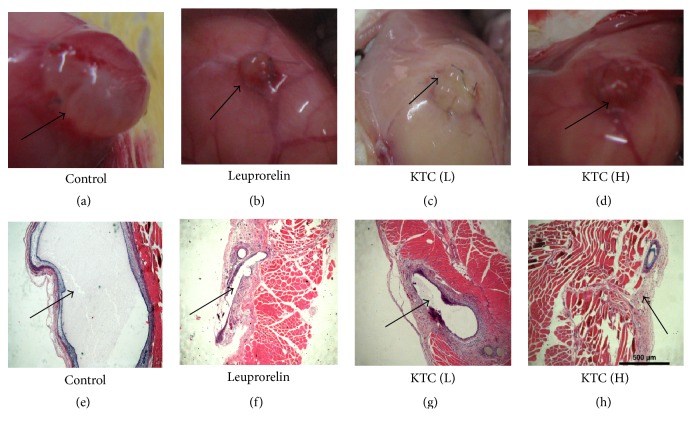
Morphological changes in ectopic endometrium after KTC treatment for 21 days. (a)–(d) Gross morphology; (e)–(h) histology (H&E, 40x). The arrows show the gross appearance and histological changes of endometrial tissues after KTC treatment.

Morphologically, endometriotic tissues appeared cystic and presented a rich vascular supply. There was no significant difference on the volume of autografts among all groups before medication and solvent treatment. At the end of treatment, there were no pronounced changes observed on the volume of endometriotic tissues in the control group compared with that of the beginning administration (*p* > 0.05, Table 2, Figure 2).

In contrast, rats treated with 0.24 and 0.6 g/kg KTC displayed 51.50 ± 21.90% and 71.97 ± 14.10% decreases, respectively, in endometriotic volumes compared with that before treatment and there was a marked difference between KTC groups and the control group (*p* < 0.05). The growth inhibitory rate of the ectopic autographs in rats treated with leuprorelin acetate was 65.60 ± 23.30%. The volumes of endometriotic tissues in leuprorelin acetate group were smaller than that in control group (*p* < 0.05, Table 2, Figure 2).

As for histology analysis, H&E staining revealed a large lumen in the ectopic endometrium in the control rats (Figure 2). After treatment with KTC and leuprorelin acetate, the implanted allografts demonstrated a much smaller lumen with degenerated epithelium. In addition, the thickness of ectopic endometrial tissues decreased to 1.29 and 0.68 mm, respectively, in KTC 0.24 and 0.6 g/kg group, significantly lower than that of control group (*p* < 0.05, Table 1, Figure 2).

### 3.3. The Effect of KTC Treatment on the Serum Levels of E_2_, Progesterone, TNF-*α*, CA125, IL-6, and COX-2

**Figure 3:**
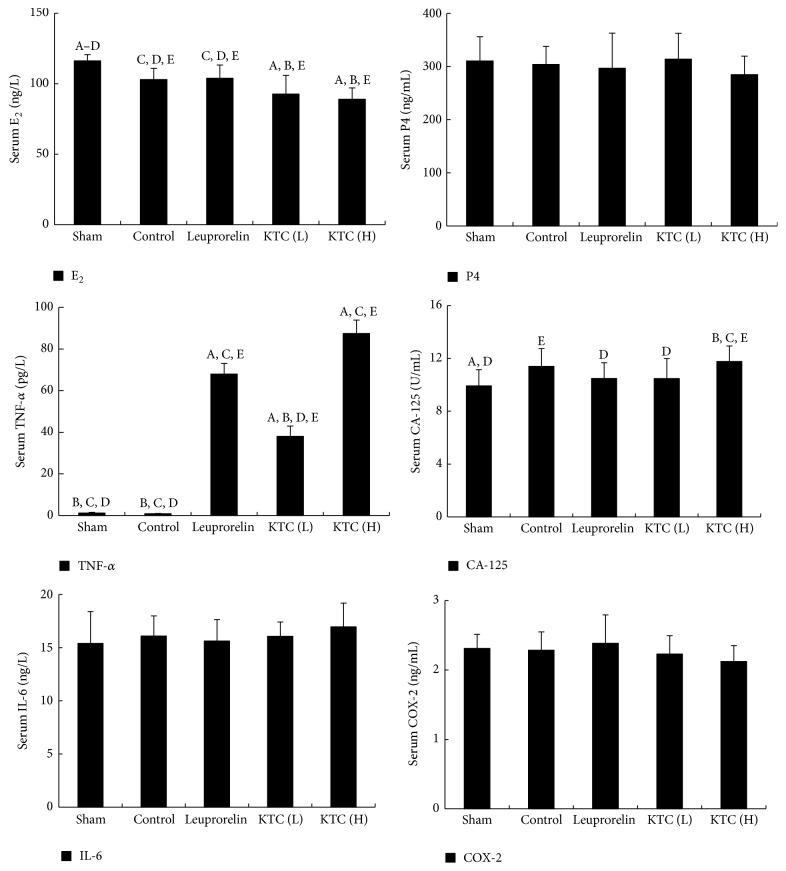
Changes of KTC on the serum levels of E_2_, progesterone, CA-125, and TNF-*α* in the rat model of endometriosis (*n* = 10 each). ^A^ *p* < 0.05, compared with the control group. ^B^ *p* < 0.05, compared with the leuprorelin group. ^C^ *p* < 0.05, compared with KTC (L). ^D^ *p* < 0.05, compared with KTC (H); ^E^ *p* < 0.05, compared with sham-operated group.

The serum levels of CA125 in the control group were higher than that of the sham-operated group (*p* < 0.05, Figure 3). There was no significant difference in the serum levels of E_2_, progesterone, TNF-*α*, IL-6, and COX-2 between the control group and the sham-operated group (*p* > 0.05, Figure 3).

Compared with the control group, the serum levels of E_2_ were significantly reduced and the levels of TNF-*α* remarkably increased in rats treated with both 0.24 and 0.6 g/kg KTC treatment groups (*p* < 0.05, Figure 3). However, there was no significant alteration observed in the serum levels of progesterone, CA125, IL-6, and COX-2 in both 0.24 and 0.6 g/kg KTC treatment groups compared with the control group (*p* > 0.05, Figure 3).

In the leuprorelin acetate treated group, the serum levels of E_2_ markedly decreased and TNF-*α* increased than those in the control group (*p* < 0.05, Figure 3). There were no distinct changes in the serum levels of progesterone, CA125, IL-6, and COX-2 in the leuprorelin acetate treated group compared with that in the control group (*p* > 0.05, Figure 3).

### 3.4. KTC Promoted Apoptosis in Ectopic Endometrial Tissues

**Figure 4:**
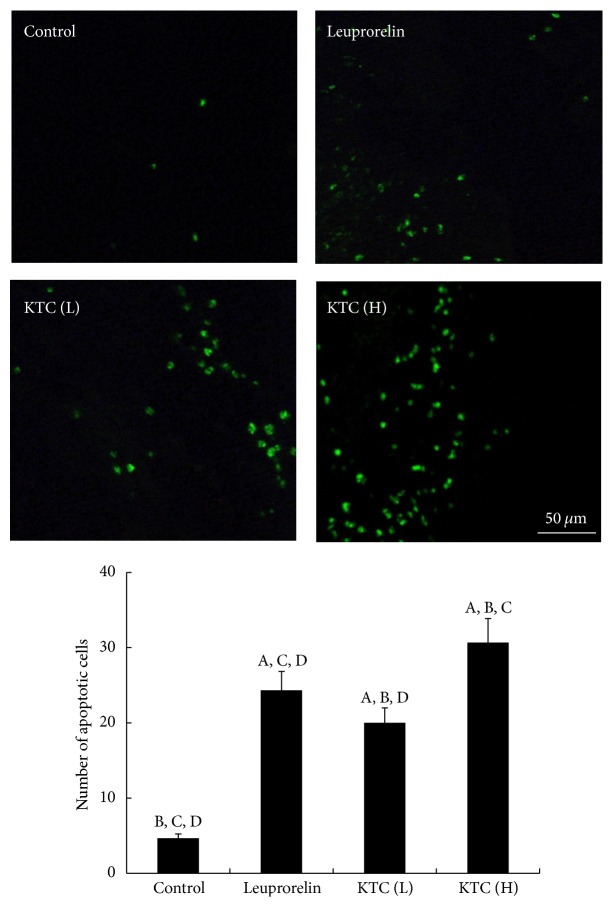
Effect of KTC on apoptosis of endometriotic tissues, as determined by the transferase-mediated digoxigenin-dUTP nick-end labeling (TUNEL) assay (×200, *n* = 3 each). Green fluorescent signals stood for the nuclei of positively stained apoptotic cells. ^A^ *p* < 0.05, compared with the control group. ^B^ *p* < 0.05, compared with the leuprorelin group. ^C^ *p* < 0.05, compared with KTC (L). ^D^ *p* < 0.05, compared with KTC (H).

As shown in Figure 4, the nuclei of positive staining apoptotic cells emitted green fluorescent signals in the ectopic endometrium tissues. The green fluorescent signals in the control group were weak (Figure 4 control). Compared to that in the control group, the numbers of apoptotic cells in the leuprorelin acetate (Figure 4 leuprorelin) and KTC groups (Figure 4 KTC (L) and KTC (H)) markedly increased. Furthermore, KTC induced apoptosis of ectopic endometrial cell in a dose-dependent manner.

### 3.5. The Effect of KTC Treatment on Protein Levels of Caspase-8, Caspase-9, Caspase-3, and Cytochrome c in Ectopic and Eutopic Endometrial Tissues

**Figure 5:**
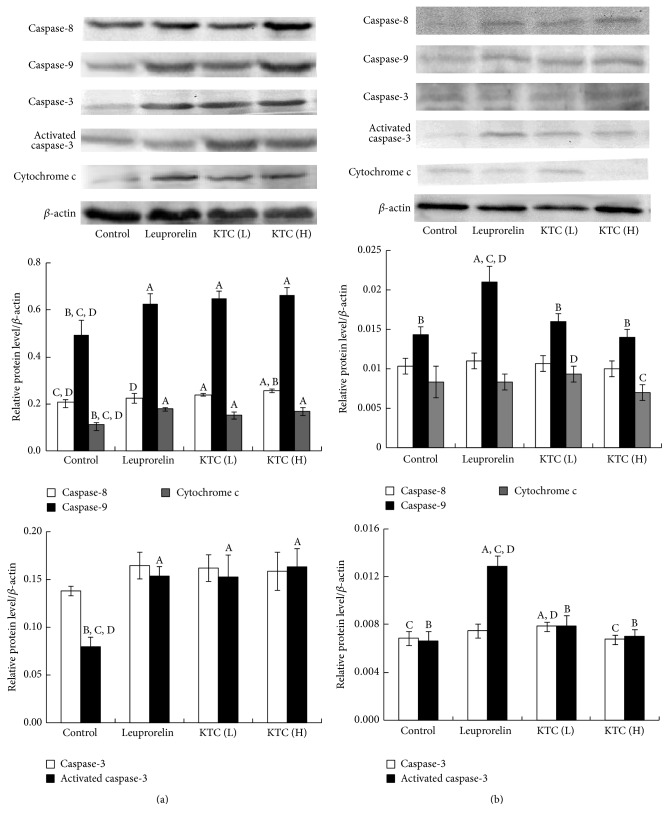
Protein levels of caspase-8, caspase-9, caspase-3, activated caspase-3, and cytochrome c in ectopic endometrial tissue and eutopic endometrial tissues in rats (*n* = 5). (a) Protein levels in ectopic endometrium. (b) Protein levels in entopic endometrium. ^A^ *p* < 0.05, compared with the control group. ^B^ *p* < 0.05, compared with the leuprorelin group. ^C^ *p* < 0.05, compared with KTC (L). ^D^ *p* < 0.05, compared with KTC (H).

Firstly, we assayed the protein levels of caspase-8, caspase-9, caspase-3, and cytochrome c in ectopic endometrial tissues via western blotting. Compared with the control group, the protein levels of caspase-8, caspase-9, and activated caspase-3 manifested a statistically significant increase in both 0.24 and 0.6 g/kg KTC treatment groups with a dose-dependent manner (*p* < 0.05, Figure 5(a)). However, the protein expressions of caspase-3 were very low and did not differ from that of the control group (*p* > 0.05, Figure 5(a)). In the leuprorelin acetate treated group, the protein levels of caspase-9 and cytochrome c remarkably increased compared with those in the control group (*p* < 0.05, Figure 5(a)). However, the protein level of caspase-8 and caspase-3 had no significant difference compared with the control group (*p* > 0.05, Figure 5(a)).

After that, we further examined these protein levels in eutopic endometrium. We found that the levels of caspase-8, caspase-9, caspase-3, and cytochrome c were markedly decreased in the eutopic endometrium (*p* < 0.05; Figures 5(a) and 5(b)) compared with those in ectopic endometrium. Furthermore, we also found that there were no significant changes in the levels of caspase-8, caspase-9, caspase-3, activated caspase-3, and cytochrome c in eutopic endometrium after treatment with both 0.24 and 0.6 g/kg KTC compared with the control group (*p* > 0.05), as shown in Figure 5(b). However, the protein levels of caspase-9 and activated caspase-3 significantly increased in rats treated with leuprorelin acetate compared with those in the control group (*p* < 0.05).

## 4. Discussion

In the present study, we found that KTC had the ability to reduce the growth of transplanted endometriotic tissues in the rat model of endometriosis. Moreover, KTC markedly induced apoptosis in the ectopic endometrial tissues through raising the protein levels of caspase-8, caspase-9, activated caspase-3, and cytochrome c, which could partially explain its therapeutic effects.

Although the rat has no menstruation and it was difficult to grow spontaneously endometriosis in rat, the endometrium in the SD rat is sensitive to hormone stimulation [25] and estrogen provides an essential dependency for the growth of ectopic endometrium [22]. Moreover, the regular endometrial cycle in rats is also similar to that in women [26]. Therefore, rat is a relatively ideal animal model for establishing model of endometriosis. Early in 1985, researchers had established a classical endometriosis model via surgical transplantation to evaluate the efficacy of antiendometriosis candidates [27, 28]. We had applied this model to confirm the effectiveness of biodegradable microspheres containing nomegestrol acetate on suppressing the growth of endometriotic tissues in previous study [21]. Here, we further utilized this model to investigate whether KTC could inhibit the proliferation of ectopic autografts. For the first time, we found that 0.24 and 0.6 g/kg of KTC remarkably inhibited the growth of endometriotic tissues in a dose-dependent manner and the average growth inhibitory rates of the endometriotic tissues are up to 51.50% and 71.97%, respectively. Moreover, we also demonstrated that KTC did not suppress the growth of eutopic endometrium inside the uterine cavity of the rat model. It indicates that KTC specifically targets the endometriotic tissues with minimal side effects on normal endometrium.

Leuprorelin acetate is an effective standard medication used to relieve endometriosis-related pain and regress the proliferation of the endometriotic tissue [29, 30]. Therefore, we gave the endometriosis rats leuprorelin acetate as a positive control to verify the experiment's reliability. Indeed, we observed that leuprorelin acetate inhibited the growth of endometriotic tissues and the average growth inhibitory rates of the endometriotic tissues are up to 65.60%. Meanwhile, the differences between the Chinese herb medicine and leuprorelin acetate were also identified. We discovered that there was no significant change on the rat's body weight in both KTC 0.24 and 0.6 g/kg treated group compared with that in control group and all animals in KTC treated groups demonstrated regular estrus cycle. However, the rat's body weight treated leuprorelin acetate in rats increased markedly. Furthermore, the results of vaginal smear showed that leuprorelin acetate influenced the estrous cycle of the rat which was at diestrus. This result implies that KTC has lesser influence on body weight than leuprorelin acetate does.

Endometriosis is an estrogen-dependent disease. Estrogen plays a key role in the pathogenesis and development of endometriosis [31]. Herein, we detected the sera concentration of E_2_ after treatment with KTC. We found that the serum levels of E_2_ obviously decreased in both 0.24 and 0.6 g/kg KTC treated group compared to that in the control group. It indicates that KTC inhibiting the proliferation of endometriotic tissues may associate with decreasing the serum level of E_2_. We also observed that leuprorelin acetate significantly reduced serum levels of E_2_. This result was consistent with previous report [32]. Besides, there were no statistically significant differences on the serum levels of progesterone among all groups. It suggests that KTC might have no significant influence on the levels of progesterone in the rat model of endometriosis.

The association between endometriosis and elevated serum CA125 levels was discovered more than thirty years ago. In Spaczynski and Duleba's meta-analysis, the level of serum CA125 was a good marker for diagnosing moderate to severe endometriosis [33], but it is unsatisfactory for mild endometriosis in sensitivity and specificity for endometriosis [34]. In the present study, we found that serum CA125 in the control group significantly increased compared with that in sham-operated group. However, the levels of serum CA125 did not remarkably reduce in KTC 0.24 and 0.6 g/kg groups compared with that in the control group. These results suggest that CA125 might not appreciably respond to KTC treatment. The result was similar to that reported previously in which nomegestrol acetate, a potent progestin, did not reduce the level of serum CA-125 in the rat model of endometriosis model [21]. In addition, we found that there was no significant change in the serum level of CA125 in the rat treatment with leuprorelin acetate. Matalliotakis et al. [35] discovered that leuprorelin acetate decreased the level of serum CA125 after three months of treatment. The disparity may be caused by the different length of treatment.

Cytokines play important roles in the process of pain. It has been reported that TNF-*α* has ability of activating COX-2 via the IL-1*β*, IL-6, and IL-8 cascades. COX-2 then promotes synthesis of prostaglandin E_2_ (PGE_2_) [36, 37]. A recent study has shown that the expression level of COX-2 was higher in ectopic lesions than in the normal endometrium [38]. In our present study, the serum levels of COX-2 and IL-6 in 0.24 and 0.6 g/kg treated groups did not significantly reduce. The result suggested that COX-2 and IL-6 may not respond to KTC treatment. In addition, we found that the serum levels of COX-2 and IL-6 have no significant fluctuation in the rat model of endometriosis after leuprorelin acetate treatment, which is different from that in human patients. Further research is necessary to verify whether KTC has effect on other inflammatory agents.

Since KTC treatment caused the shrinkage of ectopic tissues, we speculated that KTC might induce apoptosis of the endometriotic tissues. We employed the TUNEL assay to detect the apoptotic cells in the endometriotic tissues and found that KTC induced apoptosis in a dose-dependent manner. Meanwhile, we discovered that the serum levels of TNF-*α* increased after treatment with KTC. It was well known that TNF-*α* could bind TNF receptor 1 (TNFR1), promoting apoptosis through activating downstream caspases signaling proteins. The type I apoptosis pathway is initiated by the activation of death receptors followed by activation of caspase-8, which subsequently activates caspase-3, the executor of apoptosis [39]. The type II apoptosis pathway is triggered by activated caspase-8, which subsequently prompts Bid to break into tBid. tBid is then transferred into the mitochondria, leading to the release of cytochrome c into the cytoplasm. An apoptosome is formed by cytochrome c, apoptotic protease-activating factor-1 (Apaf-1), dATP, and procaspase-9 [40], resulting in activation of procaspase-9 and subsequently caspase-3. Activated caspase-3 has the ability to activate caspase-8, creating a positive feedback loop [41]. In the present study, we found that KTC increased not only the serum level of TNF-*α*, but also the protein levels of caspase-8, caspase-9, caspase-3, and cytochrome c in ectopic endometrium. It implies that KTC might induce ectopic endometrium apoptosis at the dose of 0.24 and 0.6 g/kg through increasing the levels of the above proteins in a dose-dependent manner. Interestingly, these indicators have no significant fluctuation in eutopic endometrial tissues. This result suggests that KTC causes cell death in the ectopic endometrium but spares the entopic endometrium.

It is reported that leuprorelin acetate and its analogs could increase protein levels of the activated caspase-3 and cytochrome c compared with control group in the rat model of endometriosis [42, 43]. In present study, we also found the same outcome. This result also suggests that KTC inhibiting the growth of the ectopic endometrium mechanism is similar to GnRH analogs.

In summary, the above results demonstrate that KTC could inhibit the growth of endometriotic tissue associated with apoptosis pathway through raising the protein level of caspase-8, caspase-9, activated caspase-3, and cytochrome c. Unlike leuprorelin acetate, KTC does not significantly influence the rat's body weights and regular estrus cycles. It is likely safer than leuprorelin acetate. KTC may be a promising traditional Chinese medicine to treat endometriosis. Further clinical trials are needed to confirm its efficacy in the future.

It also should be noticed that endometriosis was also associated with inflammation [44]. It has been reported that there were various components in the KTC formula. It has already been reported that some of them such as 2,5-dihydroxyacetophenone isolated from rehmanniae radix preparata could inhibit inflammatory responses [45]. In addition, white peony root has also demonstrated an activating immune effect [46]. However, the serum levels of TNF-*α* were increased in 0.24 and 0.6 g/kg treated groups. Therefore, further research is also necessary to clarify whether KTC inhibits the growth of ectopic endometrium through inhibiting inflammatory responses and regulating immunostimulatory effect.

## 5. Conclusion

Our findings suggest that KTC could inhibit the growth of ectopic endometrial tissue via upregulating the level of serum TNF-*α*, E_2_, and its downstream signaling, including the protein level of caspase-8, caspase-9, activated caspase-3, and cytochrome c.

## Figures and Tables

**Table 1:** Groups and administration.

Group	Number of rats	Drug	Dose (mg/kg)	Mode of administration
Sham	14	Solvent	—	Gavage
Control	14	Solvent	—	Gavage
Leuprorelin	14	Leuprorelin	0.01	Subcutaneous injection
KTC (L)	14	KTC	240	Gavage
KTC (H)	14	KTC	600	Gavage

**Table 2:** Inhibition of ectopic endometrium by KTC (*n* = 14, mean ± SD).

Group		Dose (mg/kg)	Volume of allografts (mm^3^)		Endometrial height (mm)	% inhibitory
			Posttreatment	Pretreatment		
1	Sham	—	—	—	—	—
2	Control	—	72.21 ± 60.23	70.86 ± 70.64	2.58 ± 0.21^B,C,D^	−9.61 ± 44.43^B,C,D^
3	Leuprorelin	0.01	21.49 ± 36.82	71.69 ± 86.52	0.63 ± 0.31^A^	65.60 ± 23.30^A^
4	KTC (L)	240	41.44 ± 39.90	71.33 ± 58.93	1.29 ± 0.38^A^	51.50 ± 21.90^A^
5	KTC (H)	600	14.78 ± 17.41	69.00 ± 84.13	0.68 ± 0.48^A^	71.97 ± 14.10^A^

^A^ *p* < 0.05, compared with the control group, the same variable. ^B^ *p* < 0.05, compared with the leuprolide group. ^C^ *p* < 0.05, compared with KTC (L) group. ^D^ *p* < 0.05, compared with KTC (H) group.
